# Factors influencing medical students’ decision to pursue a career in obstetrics and gynaecology

**DOI:** 10.1371/journal.pone.0288130

**Published:** 2023-12-05

**Authors:** Caoimhe Ní hÉalaithe, Aoife Howard, Paul Corcoran, Claire M. McCarthy, Mary Horgan, Deirdre Bennett, Keelin O’Donoghue, Suzanne O’Sullivan

**Affiliations:** 1 Department of Obstetrics and Gynaecology, University College Cork, Cork, Ireland; 2 National Perinatal Epidemiology Centre, University College Cork, Cork, Ireland; 3 Cork University Maternity Hospital, Wilton, Cork, Ireland; 4 Royal College of Physicians of Ireland, Dublin, Ireland; 5 Medical Education Unit, School of Medicine, University College Cork, Cork, Ireland; 6 Irish Centre for Maternal and Child Health Research (INFANT), University College Cork, Cork, Ireland; National Academy of Medical Sciences, NEPAL

## Abstract

**Introduction:**

The career intentions of medical students can exert influence on service provision and medical staffing in the health services. It is vital for a specialty’s development and sustainability that it has a constant stream of trainees into it annually. An appreciation of how a specialty is viewed by medical students can be used as an opportunity for early intervention in order to improve perception of the specialty and reduce future workforce problems, such as retention and attrition within obstetrics and gynaecology (O&G). We aimed to analyse positive and negative factors of the specialty of O&G as perceived by medical students in order to gain insight into changes that need to be made to improve recruitment and retention into the specialty.

**Methods:**

A 70-item structured questionnaire consisting of demographic information and 5-point Likert scale questions relating to O&G was administered to final year medical students in the Republic of Ireland. Data were analysed with descriptive statistics, logistic regression, and odds ratios as appropriate.

**Results:**

Of 195 medical students approached, 134 completed the questionnaire, a response rate of 68.7%. The majority were female (55.2%, n = 74) and 76.1% of respondents (n = 102) were Direct Entry Medicine students, with the remainder Graduate Entry Medicine students. 30.8% (n = 41) of students who responded scored 6 or more on a 10-point Likert scale when asked about their likelihood of considering a career in O&G. Students’ clerkship experience factored heavily into their perception of the specialty and was more likely to be positive if they experienced direct consultant engagement and the opportunity for hands-on experience. Lifestyle factors, litigation and media were found to be deterrents to considering the specialty after graduation.

**Conclusions:**

This study demonstrates the importance of good clerkship experience in fostering an interest amongst undergraduates in O&G. Educators and those working within the specialty should showcase the strengths of the specialty during undergraduate education, and work on ameliorating deterrents to ultimately provide a structured approach to improving recruitment into O&G.

## Introduction

Medical students are “the future of medicine”. The importance of recruitment into medical specialities should not be underestimated as it is crucial to its enhancement and sustainability [1]. Moreover, it is a vital factor for workforce planning [2]. Understanding factors that influence medical students to pursue a career in obstetrics and gynaecology (O&G) is important to predict future workforce staffing and shortages, and their implications for service provision [3]. It is important to appreciate how a medical specialty is viewed as a career opportunity by students as it creates a ‘window of opportunity’ for early intervention in order to improve perception of the specialty, encourage recruitment and reduce future strain on medical personnel [4].

By 2030, detailed workforce planning forecasts from the United States and Australia predict that there will be physician-to-population deficits in O&G of up to 20% [5, 6]. A 2018 study by the Irish Health Service Executive (HSE) National Doctors Training and Planning (NDTP) found that the employment of consultants not on the specialist divisions of the register was related to challenges with recruitment and retention of doctors [7]. O&G was one of the most challenged specialties in this regard in acute hospitals, alongside intensive care and emergency medicine. The 2018 Workforce Report by the Royal College of Obstetricians and Gynaecologists (RCOG) noted typical attrition rates from O&G training programmes of 30%, despite a predicted increased need for consultants in O&G by 2021 [8]. Among the causes for such high attrition rates are lack of team structure, undermining and bullying as well as poor work-life balance [8].

Schwartz *et al*., first coined the term “controllable lifestyle factors” in 1990 [9]. The proclivity of doctors to pursue a specialty was reported based primarily on three factors; number of hours worked weekly, frequency of nights on-call and time available for the pursuit of pastimes outside of work; O&G has long been deemed a ‘non-controllable lifestyle’ specialty [9]. Several years later, Newton *et al*., again highlighted the influence of lifestyle on medical students’ career pursuits [10].

Studies have identified factors that influence the likelihood of medical students embarking on a career in O&G, which centre predominantly on lifestyle, gender, clerkship experience and the doctors’ own enthusiasm and commitment to the specialty [11–13]. In fact, alongside surgery, O&G has been categorised as ‘most lifestyle unfriendly’ by medical students [10]. Further, it has become increasingly evident that the tendency for medical students to prioritise lifestyle when choosing a career has impacted on O&G [14]. The majority of medical students are of ‘Generation Z’ (i.e. born after 1995) and as such, pre-empting the particular preferences of this group to prioritise a work-life balance should be factored into workforce planning [15]. Recent decades have seen a large shift away from previously traditional career motivators of prestige and remuneration, towards controllable lifestyle factors [16]. In a 1995 longitudinal British Medical Association (BMA) cohort study [12], 5% of graduating doctors had plans for a career in O&G but this had fallen to 1% by 2002. This seems to be rising again, as O&G was the first choice of career for 5.7% of post-2002 graduates in year 1, 4.3% in year 4 and 3.8% in year 5 [12].

Recurring themes that deter doctors from pursuing a career in O&G are poor clerkship experiences, heavy workload and medical litigation, as well as difficulty in gaining hands-on experience due to the frequent reluctance of female patients to consent to history taking and intimate clinical examination [17, 18]. In a 2009 study it was reported that factors encouraging an early career choice of O&G included a positive undergraduate clerkship; both consultant and junior doctor role-models; a mix of medicine and surgery; and hands-on training opportunities [13]. Interestingly, the same cannot be said for all medical specialities as research from Devine *et al*., would suggest there is less evidence to support the influence of student clerkships on career choices, apart from those in primary care [19] A 2006 RCOG document outlined a list of undergraduate placement standards in an effort to improve the clerkship experience and reverse declining interest in the specialty, in response to strong evidence that students’ undergraduate experience impacted significantly on their attraction to the specialty [13]. Students rated these standards as highly appropriate, with a greater interest in pursuing O&G than was previously documented [20].

Current evidence confirms that the decline in numbers choosing to pursue further training in O&G is particularly marked among newly qualified male doctors [12]. It appears that as well as more difficult access to direct clinical experience at medical school, the emerging predominance of female obstetricians and gynaecologists acts as a further deterrent to male graduates, thus perpetuating the cycle and leading to more pronounced gender imbalance [20].

A study by Frattarelli et al [21] in the US which examined longitudinal integrated clerkships (LIC) found that a higher percentage of students pursued O&G residency programmes if they had participated in an LIC instead of a traditional block rotation highlighting again that diverse clerkship experiences has a positive influential effect on O&G. Lifestyle issues, the experience of clerkships and arduous training pathways are recurring elements it seems, with trainees continuing to report these deterring factors in RCOG surveys [22]. However, the extent to which these factors influence students in Ireland, and their relative degrees of importance in the decision-making process, is not well known for O&G. Career choices of medical choices may also be influenced by the structure of the healthcare system they are trained in, for example a dual state- and privately-funded system in the Republic of Ireland. The influences of recent changes, such as the introduction of the European Working Time Directive (EWTD), are not reflected in more recent international research. It is worth noting that EWTD was introduced in Ireland in 2009, and the country received an ultimatum from the European Commission in 2011 for their non-compliance with it [23]. Despite this ultimatum, the EWTD is an issue that junior doctors are still waiting to have implemented today [24]. Additionally, potential future educational approaches have not been examined which may be effective in encouraging students to see the specialty more favourably. Understanding how different factors impact the students’ perception of a specialty and which factors are modifiable, is important to encourage the next generation of obstetricians and gynaecologists in-training.

Considering the wide-ranging factors that influence medical students’ desire to pursue a career in O&G, the context and geographical location [25, 26] within which they are trained should not be underestimated. At present, the evidence does not exist to predict these factors in a population training in the Republic of Ireland. The primary aims of this study were to determine the career preferences of medical students in the Republic of Ireland in their final year of studies and to understand factors influencing the likelihood of them pursuing a career in O&G.

## Methods

Utilising an epidemiological quantitative methodology, we conducted a cross-sectional study of medical students at the outset of their final year at an Irish university. Given the lack of suitably validated questionnaires for this purpose, an extensive literature review and appraisal was carried out which led to the construction of a 70-item questionnaire (see S1 Appendix) covering five main areas:

- Demographics of participants (such as age, gender, nationality)
- Career choice
- Placement experience
- Characteristics of O&G
- Theoretical changes to O&G

For each of the aforementioned categories, students were asked to indicate their agreement or disagreement with a statement based on a 5-point Likert scale. Those who answered 1–3 were deemed not to agree with a statement, and those who answered 4–5 were deemed to agree with the statements. The questionnaire was initially piloted amongst 24 students and was refined based on their responses. A hard copy of the questionnaire was given to all final year medical students on the first day of the first semester to maximise study participation. The group consisted of students in both Direct-Entry (DEM) and Graduate-Entry Medicine (GEM), which are five- and four-year programmes respectively. Gender was recorded as a binary variable.

### Statistics

The data were analysed using IBM SPSS Statistics Version 25. Graphical summaries were used to illustrate the frequency of responses to a range of questions. Summary statistics were used to describe respondent demographics and response frequencies. The five-point levels of agreement indicated by respondents to the range of statements provided were dichotomised to allow comparison of those in agreement (agree or strongly agree) with those not in agreement (strongly disagree, disagree, neither). Likelihood of considering O&G, originally scored on a scale of 0–10 was also dichotomised. We considered those giving a likelihood of 6–10 as representing those who were more-likely-than-not to consider O&G. Cross-tabulations with Pearson chi-square tests were used to summarise and assess the associations between these dichotomised variables. Univariate logistic regression models were utilised to quantify the strength of these associations using odds ratios and their 95% confidence intervals and p-values. We applied a Bonferroni adjustment to these p-values by multiplying them by 32, which is the number of factors we considered as potentially influencing likelihood to pursue O&G. Almost all variables were completed by all 134 respondents. We performed complete-case analysis and the final multivariable model utilised data from 133 respondents.

### Ethics

Ethical approval was sought and granted from the Clinical Research Ethics Committee of the Cork Teaching Hospitals ECM 6 (cd) 06/12/16 & ECM 3 (ooo) 09/05/17. A comprehensive cover letter incorporating the study objectives accompanied each questionnaire. Written informed consent was obtained from all participants, who were free to withdraw from the study at any point prior to return of the questionnaire. Completion and return of the questionnaire was additionally taken as consent to participate.

## Results

A total of 134 completed questionnaires were collected from a class of 195 students, giving a response rate of 68.7%.

### Respondent demographics

The majority of respondents were female (55.2%; n = 74), and enrolled on the DEM programme (76.1%, n = 102). The latter reflects an over-representation of DEM students, who actually represent 62.0% of class. The median age of respondents was 23 years (range 21–37). Just over one-third (36.5%; n = 49) felt they knew what specialty they wished to pursue following graduation.

### O&G as first choice specialty

**Fig 1:**
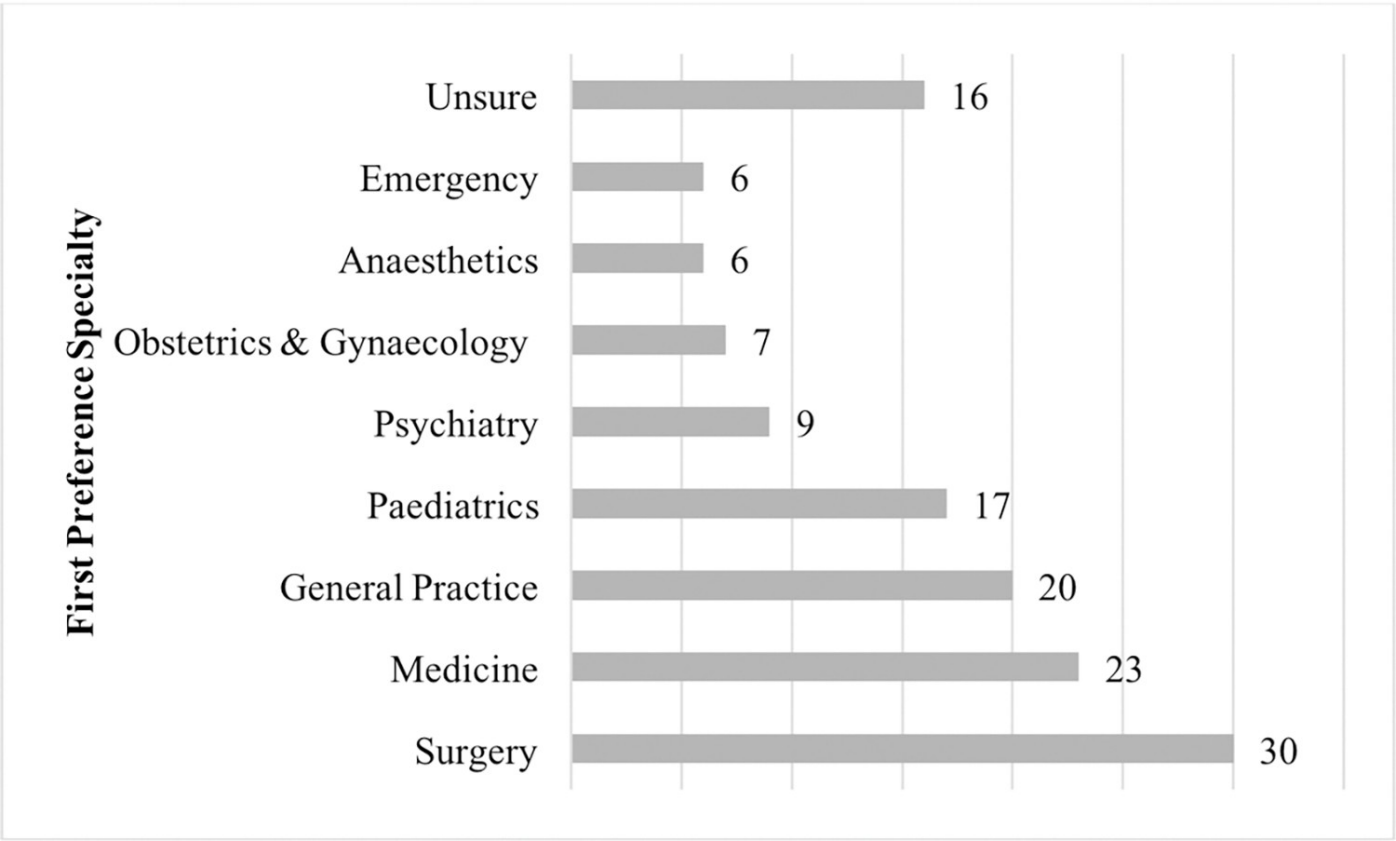
Students’ first preference specialty.

When asked what choice of specialty they may wish to pursue, 5.3% (n = 7) indicated O&G as their first choice. All were female and enrolled on the DEM programme, with three students originally from Malaysia. Fig 1 illustrates the overall spread of specialty choices, with surgery, medicine and primary care being the most popular.

Almost one third of the students indicated that they were likely to pursue O&G as a career (n = 93 of 134, 30.6%) and this was the case for male and female students (Table 1).

**Table 1:** Likelihood of pursuing obstetrics and gynaecology as a career.

	0–5 (less likely)	%	6–10 (more likely)	%
Female (n = 74)	52	70.3	22	29.7
Male (n = 59)	40	67.8	19	32.2
All (n = 134)*	93	69.4	41	30.6

*One respondent omitted gender. Chi-Square = 0.094, df = 1, crude p-value = 0.759, Bonferroni-adjusted p-value>1.

### Experience of the clinical clerkship

Two-thirds of the students (n = 88 of 134, 65.7%) agreed that their attachment increased their interest in O&G and these students were far more likely to indicate that they would pursue O&G as a career (Table 2). The odds of being likely to pursue O&G were doubled for students who agreed that they gained valuable hands-on experience, who felt satisfied with their rotation and who had positive interactions with doctors.

**Table 2:** Students’ responses to statements on clinical clerkships and the influence of their experiences on likelihood to pursue obstetrics and gynaecology as a career.

Statement on Overall Placement Experience	Agrees (Yes/ No)	Likely to pursue		OR (95% CI)	Crude p-value	Adjusted p-value*
		n	%			
I gained valuable hands-on experience	Y (n = 72)	27	37.5	2.06 (0.96–4.41)	0.064	>1
	N (n = 62)	14	22.6			
My attachment increased my interest in O&G	Y (n = 88)	36	40.9	5.68 (2.05–15.76)	<0.001	0.027
	N (n = 46)	5	10.9			
I felt satisfied with my experience of the rotation	Y (n = 81)	31	38.3	2.67 (1.17–6.06)	0.019	0.608
	N (n = 53)	10	18.9			
I had positive interactions with nurses and midwives	Y (n = 74)	21	28.4	0.79 (0.38–1.66)	0.536	>1
	N (n = 60)	20	33.3			
I had positive interactions with doctors	Y (n = 97)	34	35.1	2.31 (0.92–5.82)	0.075	>1
	N (n = 37)	7	18.9			
I had positive interactions with patients	Y (n = 118)	37	31.4	1.37 (0.41–4.53)	0.606	>1
	N (n = 16)	4	25.0			
I felt that my gender negatively influenced my learning experience	Y (n = 41)	15	36.6	1.49 (0.68–3.24)	0.319	>1
	N (n = 93)	26	28.0			
I felt that my gender positively influenced my learning experience	Y (n = 39)	11	28.2	0.85 (0.38–1.93)	0.700	>1
	N (n = 95)	30	31.6			

Note: O&G = Obstetrics and gynaecology, OR = odds ratio, CI = confidence interval. *Bonferroni-adjusted p-value

### Characteristics of O&G

**Fig 2:**
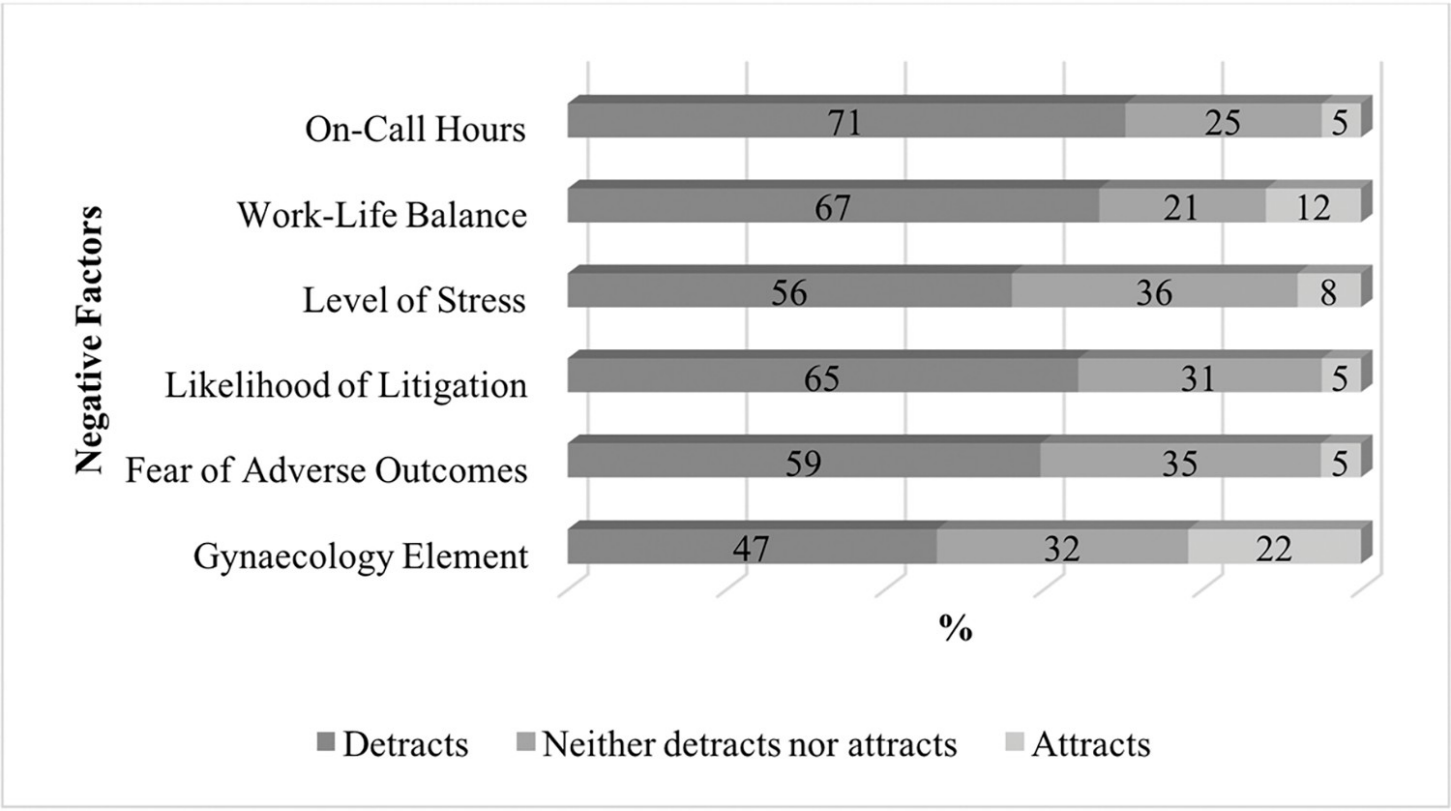
Factors that negatively influence medical students towards a career in O&G.

**Fig 3:**
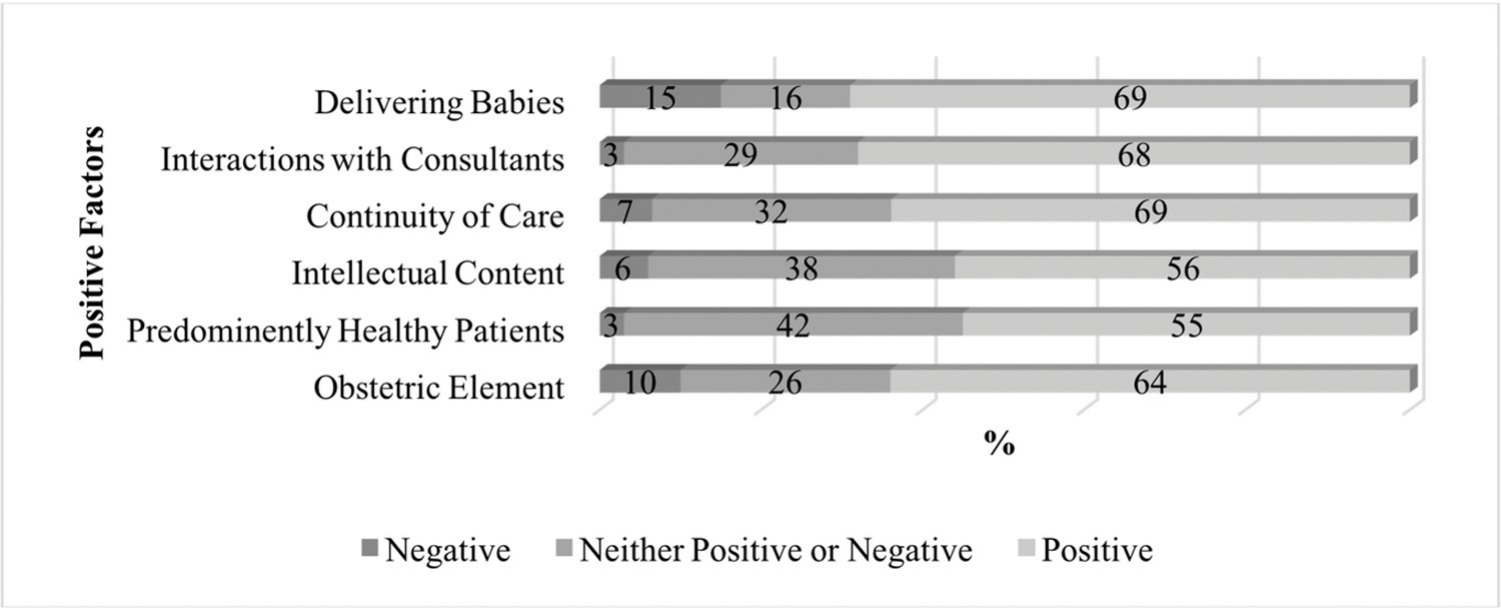
Factors that positively influence medical students towards a career in O&G.

Students were asked what impact a list of factors had on their perception of O&G as a career option on a scale of 1–5. Responses are summarised in Figs 2 and 3.

Lifestyle issues including on-call hours, work-life balance and levels of stress were reported as negative factors. The likelihood of litigation and apprehension around adverse outcomes were cited by 63.4% (n = 85) and 58.9% (n = 79) of students’ respectively as negative characteristics of O&G.

The obstetric component of the specialty was seen as attractive by 64.9% (n = 87) of survey respondents. It is clear also that obstetrics specifically, and ‘delivering babies’ remains core to the allure of the specialty. Continuity of care, and a predominantly healthy patient population, exerted a positive influence on students’ attitudes towards the specialty (61.9%; n = 83 and 54.5%; n = 73 respectively). Interactions with O&G consultants were deemed by 67.9% (n = 91) of survey participants to positively affect their views on the specialty.

Using logistic regression analysis, each factor was examined to correlate the relationship between them and students who expressed an interest in considering O&G. Table 3 lists factors that increased attraction to the specialty, while Table 4 lists those that did not. There was little to no difference between genders as regards these factors influencing their decision to pursue O&G.

**Table 3:** Factors that increased attraction to obstetrics and gynaecology as a career.

Factor	Attracts (Yes/ No)	Likely to pursue		OR (95% CI)	Crude p-value	Adjusted p-value*
		n	%			
Continuity of care	Y (n = 82)	33	40.2	4.23 (1.70–10.53)	0.002	0.064
	N (n = 51)	7	13.7			
Female patients only	Y (n = 24)	12	50.0	2.79 (1.13–6.91)	0.026	0.832
	N (n = 110)	29	26.4			
Delivering babies	Y (n = 93)	36	38.7	4.55 (1.63–12.67)	0.004	0.128
	N (n = 41)	5	12.2			
Limited focus of disease	Y (n = 56)	24	42.9	2.69 (1.27–5.72)	0.010	0.320
	N (n = 78)	17	21.8			
Intellectual content	Y (n = 76)	32	42.1	3.96 (1.70–9.21)	0.001	0.032
	N (n = 58)	9	15.5			
Combination of O&G	Y (n = 57)	24	42.2	2.57 (1.21–5.45)	0.014	0.448
	N (n = 77)	17	22.1			
Obstetric element	Y (n = 86)	33	38.4	3.11 (1.30–7.47)	0.011	0.352
	N (n = 48)	8	16.7			
Predominance of female practitioners	Y (n = 19)	12	63.2	5.08 (1.83–14.14)	0.002	0.059
	N (n = 115)	29	25.2			
Career opportunities	Y (n = 62)	27	43.5	3.20 (1.48–6.90)	0.003	0.096
	N (n = 72)	14	19.4			
Interaction with consultants	Y (n = 91)	34	37.4	3.07 (1.23–7.65)	0.016	0.512
	N (n = 43)	7	16.3			

Note: O&G = Obstetrics and gynaecology, OR = odds ratio, CI = confidence interval. *Bonferroni-adjusted p-value

**Table 4:** Factors that did not increase attraction to obstetrics and gynaecology as a career.

Factor	Attracts (Yes/ No)	Likely to pursue		OR (95% CI)	Crude p-value*
		n	%		
Level of stress	Y (n = 11)	4	36.4	1.37 (0.38–4.95)	0.636
	N (n = 122)	36	29.5		
On call hours	Y (n = 6)	2	33.3	1.14 (0.20–6.49)	0.882
	N (n = 128)	39	30.5		
Gynecology element	Y (n = 30)	10	33.3	1.18 0.49–2.80)	0.712
	N (n = 104)	31	29.8		
Fear of adverse outcomes	Y (n = 8)	3	37.5	1.39 (0.32–6.11)	0.663
	N (n = 126)	18	30.2		
Media Portrayal	Y (n = 6)	3	50.0	2.37 (0.46–12.27)	0.304
	N (n = 128)	38	29.7		
Likelihood of litigation	Y (n = 7)	1	14.3	0.36 (0.04–3.11)	0.355
	N (n = 127)	40	31.5		
Financial remuneration	Y (n = 35)	10	28.6	0.88 (0.38–2.05)	0.762
	N (n = 99)	31	31.3		
Work-life balance	Y (n = 16)	4	25.0	0.73 (0.22–2.41)	0.606
	N (n = 118)	37	31.4		
Duration of training	Y (n = 15)	5	33.3	1.15 (0.37–3.61)	0.807
	N (n = 119)	36	30.3		
Working under pressure	Y (n = 26)	10	38.5	1.55 (0.63–3.79)	0.335
	N (n = 108)	31	28.7		
Increased patient expectations	Y (n = 41)	13	31.7	1.08 (0.49–2.38)	0.883
	N (n = 93)	28	30.1		
Interaction with midwives	Y (n = 35)	11	31.3	1.05 (0.46–2.42)	0.901
	N (n = 99)	30	30.3		
Interaction with NCHDs	Y (n = 60)	20	33.3	1.26 (0.60–2.64)	0.536
	N (n = 74)	21	28.4		

*Bonferroni-adjusted p-value > 1 for all associations

A student that rated continuity of care as an attractive component was more likely to be interested in pursuing a career in O&G. Students who were attracted to delivering babies, and attracted to the intellectual content of O&G, also had four times higher odds of indicating that they were likely to pursue a career in O&G. While a relatively low number of participants (*n* = 19) saw a predominance of female practitioners as an attractive factor for O&G. It is worth noting that the odds of being likely to pursue O&G as a career was five times higher in this subset.

Table 4 lists factors where no significant correlation was found that increased the likelihood of students pursuing a career in O&G.

### Theoretical factors to increase the appeal of O&G for students

**Fig 4:**
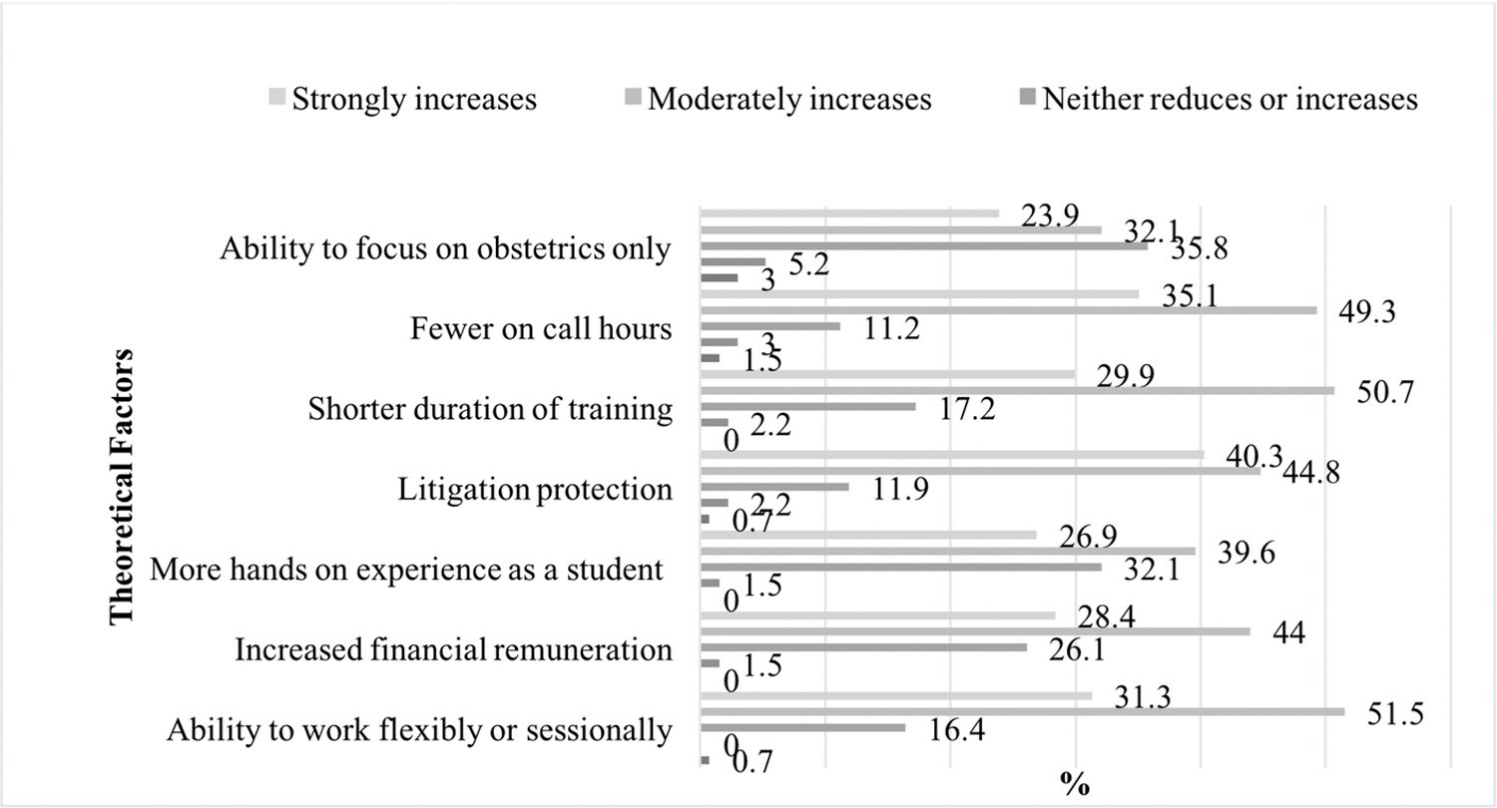
Theoretical factors that may alter the appeal of the specialty.

Students were presented with a range of theoretical changes to the specialty that would make it more alluring, as demonstrated in Fig 4. More hands-on experience on labour ward was deemed to be a potential positive change, with 39.6% (n = 53) citing it as a moderate increase in the attractiveness of O&G and 26.9% (n = 36) expressing it would strongly increase its attractiveness.

A reduced level of litigation would be the single most attractive change to O&G, with 84.3% (n = 113) of students reporting it would moderately/strongly increase the specialty’s appeal. Lifestyle factors, such as flexible/reduced working hours would make O&G a more attractive career option for up to 82.8% (n = 111) of surveyed students, in addition to reduced on-call commitments. Other factors like shorter training durations, and increased financial remuneration would increase the appeal of O&G in 79.9% (n = 107) and 72.4% (n = 97) of students surveyed respectively.

## Discussion

This study identified factors that influence medical students’ attraction to O&G within the context of one medical school in the Republic of Ireland. The data from it, while may not be generalizable outside the Republic of Ireland, may be extrapolated to inform and guide future research in the area. Clear insight into the perception of current students towards the discipline is imperative because their recruitment is key to future workforce planning. Knowledge of their clerkship experience and their perceptions of O&G as a career path should enable targeted interventions to optimise their interest and likelihood of potential recruitment.

While only a small number of students indicated O&G as their first choice specialty, nearly one third would potentially consider a career in O&G. Despite there being little overall difference in findings between genders throughout the study, only female respondents indicated that O&G was their chosen specialty. Bienstock and Laube found that male students attribute part of their hesitancy to enter the specialty to their experience of clinical rotations [27], but we found no significant difference between male and female participants in their perception of whether their gender influenced this.

O&G incorporates a wide breath of clinical practice with many opportunities to subspecialise in areas of interest. The 2003 RCOG Trainees Committee survey highlighted the increasing consensus that the specialty should be divided into separate disciplines with 70% of respondents in favour of the separation [28], which was also expressed in a 2006 survey [29]. This was reflected in our survey, with over half of students of the opinion that a sole obstetric focus would be more appealing. The fact that specialty division may lead to a theoretical shorter duration of training was also attractive to most respondents.

Delivering babies, intellectual content, and continuity of care were aspects of the discipline that all significantly positively impacted students’ attraction to a career in the specialty in this study. Perception of career opportunities also had a significantly positive influence on respondents. It was clear that aspects of obstetrics, like those mentioned above, appear central to its attraction as a career, some of which are quite unique over other areas of medicine. It is something which must not be underestimated when looking to improve attraction to the speciality at medical school.

The importance of a student’s clerkship experience in relation to their desire to pursue O&G as a career is well recognised [30], and this study supports the literature. Those having a positive experience were over 5-times more likely to consider pursuing a career in O&G. The study reflects research showing the positive effect a satisfying rotation experience can have [31], in contrast to the majority of existing research on the topic which focuses primarily on the negative effects of a poor clerkship experience. The role of consultants as role models for the future of the specialty should not be underestimated with students noting consultant interaction as integral to their clerkship experience [32].

Our respondents were also keen to gain additional ‘hands-on’ experience, which is consistent with previous studies, and advocates for more active participation during O&G rotations [33]. While still core to some medical schools’ approaches, a call for universal re-instatement of the hands-on experience traditionally associated with the rotation is needed reflecting students’ feedback in cultivating interest in the specialty [34].

The majority of respondents from this study cited that the likelihood of future litigation detracted them from O&G, and that protection from litigation would encourage this career choice, in keeping with previous literature [35]. In Ireland, this fear was also seen to persevere with trainees of the specialty pointing to a wider call for action, noting the media’s responsibility to doctors and the public when reporting clinical events [36].

This study’s findings underpin the existing classification of O&G as a ‘lifestyle unfriendly’ discipline [10]. Students are keen to prioritise ‘controllable lifestyle’ factors in their selection of specialty [37], and the lack of flexibility in O&G is a significant deterrent to recruitment. More than 70% of those surveyed in our study rated on-call hours as a detracting factor, consistent with Schnuth *et al*’s findings in 2003 [3]. While the introduction of the EWTD has somewhat ameliorated this, an RCOG report found that 88% of obstetric units still reported a gap in their middle-grade rota [8]. Despite this, those who have a genuine desire to pursue O&G are less likely to be dissuaded by the ‘unfriendly’ lifestyle [38].

Consideration needs to be given to the “Generation Z” origin of the modern-day medical students. Adaptation to their needs and aspirations, such as flexible working hours and cultural changes within the specialty, will ultimately help improve interest in the career [39]. They tend to be active problem solvers, independent learners, and advocates for social justice, equality and the environment [40]. Adapting training and working within O&G to accommodate these desires more comprehensively could serve to improve recruitment into the specialty.

An overwhelming majority of respondents of this survey agreed that a more flexible working life would serve to largely increase the appeal of O&G. The National Doctors Training and Planning (NDTP) with the Royal College of Physicians of Ireland have been proactive in promoting flexible training opportunities and part-time consultancy positions in recent years, although opportunities remain limited in numbers. Data from the UK supports altering the traditional contract structure in order to optimise recruitment and retention [41]. The perceived culture of inflexible lifestyles is slowly changing, in keeping with recommendations from the Irish Government’s Strategic Review of Medical Training and Career Structure 2017–2018 [42].

The thorough structure of this questionnaire, completed by an invested group of students is a key strength of this study, giving a multi-factorial insight into career determinants and direction of future medics. It is worth noting that students were presented with a series of ideas rather than given the opportunity to present their thoughts without prompting, this is a possible limitation that could be improved upon in future studies. We also propose potential solutions that might increase the appeal of the specialty to students, informing future approaches to workforce planning in Ireland. While the relatively high response rate is a strength of the study, being set in one university and affiliated hospital, rather than a national setting, is a limitation. It may be generalizable within the Republic of Ireland, but it is difficult to say whether it would be generalizable in the context of differing demographics, clerkships, debt load on students and trainees etc. Demographics of the respondents and non-respondents within the sample cohort were not compared. It is also worth noting that in the context of this voluntary survey it is likely a response bias was present, that is to say those with strong interests in pursuing O&G, and conversely those with no interest in pursuing it, were more likely to respond or have strong opinions either way.

## Conclusion

To conclude, this study shows that only a small number of Irish medical students sampled plan to choose O&G as their practising specialty into the future. We highlight the importance of clerkship experience and role models in fostering an early interest in O&G at undergraduate level. As such, it is imperative that the strengths of the specialty are highlighted. The greater understanding of perceived deterring factors unearthed in this study should be used in a streamlined way to showcase the changing culture that is emerging in the specialty as well as provide food-for-thought on improving attraction towards O&G.

## Supporting information

S1 Appendix (DOCX)
