# Supplementary material for: Factors influencing medical students’ decision to pursue a career in obstetrics and gynaecology
Source: PLoS One. 2023 Dec 5;18(12):e0288130. doi: 10.1371/journal.pone.0288130 (PMC10697605; doi:10.1371/journal.pone.0288130)
Supplement: S1 Appendix — (DOCX) [file pone.0288130.s001.docx]

**Appendices**

1. **Questionnaire for medical students**

Questionnaire

Age:

Gender; Female

Male

Class;

Direct entry medicine year 5

Graduate entry medicine year 4

Nationality:

Do you have children? Yes

No

**Career choice**:

I know what specialty I wish to pursue after graduation: Yes

No

On a scale of 1 to 10 please rate the likelihood of you pursuing a career in Obstetrics and Gynaecology where 1 represents a definite no and 10 a definite yes;

Please state your first preference speciality in the space provided;

**Overall placement experience:**

Below are a series of statements pertaining to your Obstetrics and Gynaecology rotation please indicate whether you agree with these by using a scale from 1 to 5. The scale is as follows.

1; completely disagree, 2; moderately disagree, 3; no affect, 4; moderately agree 5; completely agree.

Please place an X in the relevant column.

| Statement | 1  Completely disagree | 2  Moderately disagree | 3  No effect | 4  Moderately agree | 5  Completely agree |
| --- | --- | --- | --- | --- | --- |
| I gained valuable hands on experience |  |  |  |  |  |
| My attachment increased my interest in OB/GYN |  |  |  |  |  |
| I felt satisfied with my experience of the rotation |  |  |  |  |  |
| I had positive interactions with nurses + midwives |  |  |  |  |  |
| I had positive interactions with doctors |  |  |  |  |  |
| I had positive interactions with patients |  |  |  |  |  |
| I feel that my gender negatively influenced my learning experience |  |  |  |  |  |
| I feel that my gender positively influenced my learning experience |  |  |  |  |  |

**Characteristics of obstetrics and** **gynaecology**:

This section contains a series of statements relating to OB/GYN, please select whether or not you believe that the factors listed below attract or detract you as a medical student to/from the speciality or are of no influence using a scale of 1 to 5. The scale is as follows;

1; strongly detracts me from OB/GYN 2; moderately detracts 3; no effect, 4; moderately attracts, 5; strongly attracts me to OB/GYN

Please place an X in the relevant column.

| Factors | 1  Strongly detracts | | 2  Moderately detracts | 3  No effect | 4  Moderately attracts | 5  Strongly attracts |
| --- | --- | --- | --- | --- | --- | --- |
| Predominantly healthy patient population | |  |  |  |  |  |
| Continuity of care |  | |  |  |  |  |
| Female patients only |  | |  |  |  |  |
| Carrying out surgery |  | |  |  |  |  |
| Carrying out deliveries |  | |  |  |  |  |
| Limited focus of disease |  | |  |  |  |  |
| Intellectual content of OB/GYN |  | |  |  |  |  |
| Combination of obstetrics and gynaecology |  | |  |  |  |  |
| On call hours |  | |  |  |  |  |
| Gynaecological element |  | |  |  |  |  |
| Obstetric element |  | |  |  |  |  |
| Predominance of female OB/GYNs |  | |  |  |  |  |
| Career opportunities |  | |  |  |  |  |
| Current debate regarding abortion |  | |  |  |  |  |
| Fear of adverse outcomes |  | |  |  |  |  |
| Media portrayal |  | |  |  |  |  |
| Likelihood of litigation |  | |  |  |  |  |
| Financial remuneration |  | |  |  |  |  |
| Work/ life balance |  | |  |  |  |  |
| Duration of training |  | |  |  |  |  |
| Level of stress |  | |  |  |  |  |
| Working under pressure |  | |  |  |  |  |
| Increasing patient expectations e.g. positive birth experiences |  | |  |  |  |  |
| Interactions with midwives on placement |  | |  |  |  |  |
| Interactions with NCHDs |  | |  |  |  |  |
| Interactions with consultants |  | |  |  |  |  |

**Would you be more likely to consider OB/GYN if?**

The following section contains a series of statements referring to theoretical changes in OB/GYN please select whether you believe these changes would make the speciality either more appealing, less appealing or be of no effect using a scale from 1 to 5. The scale is as follows;

1; strongly reduces appeal, 2; moderately reduces appeal, 3; no effect, 4; moderately increases appeal, 5; strongly increases appeal.

Please place an X in the relevant column.

| Change | | | 1  Strongly reduces | 2  Moderately reduces | 3  No effect | | 4  Moderately increases | | 5  Strongly increases |
| --- | --- | --- | --- | --- | --- | --- | --- | --- | --- |
| Ability to concentrate on obstetrics only | | |  |  |  | |  | |  |
| Ability to concentrate on gynaecology only | | |  |  |  | |  | |  |
| Ability to focus solely on surgical side | | |  |  |  | |  | |  |
| Ability to focus solely on medical side | | |  |  |  | |  | |  |
| Fewer on call hours | | |  |  |  | |  | |  |
| Shorter duration training (currently min.8 years) | | |  |  |  | |  | |  |
| Litigation protection | | |  |  |  | |  | |  |
| More exposure to OB/GYN as a student. | | |  |  |  | |  | |  |
| More hands on experience as a student e.g. labour ward | | |  |  |  | |  | |  |
| Increased financial remuneration | | |  |  |  | |  | |  |
| Ability to work flexibly as opposed to a “full” consultant with on call |  |  | |  | |  | |  | |

**Media portrayal of obstetrics and gynaecology:**

This section contains a series of statements examining the portrayal of OB/GYN in the media, you are asked to state whether you agree or disagree with these using a scale from 1 to 5. The scale is as follows:

1; completely disagree, 2; moderately disagree, 3; unsure, 4; moderately agree 5; completely agree.

Please place an X in the relevant column.

| Statement | | | 1  Completely disagree | | 2  Moderately disagree | 3  Unsure | 4  Moderately agree | | 5  Completely agree |
| --- | --- | --- | --- | --- | --- | --- | --- | --- | --- |
| OB/GYN is positively portrayed in the media | | |  | |  |  |  | |  |
| The media portrayal of OB/GYN has influenced my attitude towards it as a potential career | | |  | |  |  |  | |  |
| The media portrayal of OB/GYN has made me less likely to pursue a career in it | | |  | |  |  |  | |  |
| OB/GYN as a specialty is the subject of more media focus than other specialities | | |  | |  |  |  | |  |
| Media portrayal of OB/GYN has increased my fear of adverse outcomes | | |  | |  |  |  | |  |
| Media portrayal of OB/GYN has increased my perception of litigation | | |  | |  |  |  | |  |
| Media portrayal has influenced patients attitude towards OB/GYN doctors in a negative way |  |  | |  | |  | |  | |

Do you have any more thoughts on this subject?

Thank you for your time and participation in this study.
